# Case Report: Distal ureteral obstruction decades after presumed intrauterine device removal in a woman with uterus didelphys

**DOI:** 10.3389/fmed.2026.1985408

**Published:** 2026-10-07

**Authors:** Chengsheng Xiao, Cheng Li

**Affiliations:** 1 Department of Urology, Changde Hospital, Xiangya School of Medicine, Central South University (The First People’s Hospital of Changde City), Changde, China; 2 Department of Obstetrics and Gynecology, Changde Hospital, Xiangya School of Medicine, Central South University (The First People’s Hospital of Changde City), Changde, China

**Keywords:** case report, ectopic intrauterine device, intrauterine device, ureteral obstruction, uterus didelphys

## Abstract

**Background:**

An ectopic intrauterine device (IUD) can rarely cause ureteral obstruction and hydroureteronephrosis. Diagnosis may be delayed when a prior removal procedure is believed to have been complete.

**Case presentation:**

A 48-year-old woman presented with a 1-year history of right flank pain and urinary frequency after repeated treatment for presumed urinary tract infection and possible genitourinary tuberculosis without sustained improvement. More than 20 years earlier, an IUD had been inserted before uterus didelphys was recognized. Two to three years later, an unintended pregnancy led to recognition of the duplicated uterine anatomy, induced abortion, and an IUD removal procedure. The original records were unavailable, so device location and intact retrieval at that time could not be verified. Multimodality imaging at the index admission showed severe right hydroureteronephrosis, focal distal ureteral narrowing, and an ectopic metallic IUD outside both uterine cavities; cystoscopy was unremarkable. Open ureteral exploration and reconstruction identified an intact IUD with an attached calculus externally compressing an approximately 1-cm fixed distal ureteral stricture without entering the ureteral lumen. The narrowing persisted after device removal, requiring segmental ureteral resection and anastomosis over a double-J stent. Histopathology of the resected ureter showed chronic inflammatory changes without tuberculous changes. The stent was removed 2 months postoperatively. At 6 months after surgery, computed tomography showed marked decompression of the right collecting system and no residual pelvic metallic device; serum creatinine remained normal.

**Conclusion:**

A history of IUD removal should not be treated as proof of device absence when intact retrieval is undocumented. In otherwise unexplained unilateral ureteral obstruction, a remote IUD history should prompt review of prior device localization and targeted pelvic imaging, particularly when historical records are unavailable or duplicated uterine anatomy complicates localization. A fixed stricture may require reconstruction even after the ectopic device is removed.

## 1 Introduction

Intrauterine devices (IUDs) are highly effective contraceptives; however, uterine perforation, malposition, and extrauterine displacement can produce delayed complications. Urinary tract involvement is uncommon and predominantly affects the bladder. In a 2024 review of 115 reported urinary tract IUD migrations or translocations, bladder involvement predominated, whereas only seven cases involved the ureters (1). Although rare, ureteral involvement can produce hydronephrosis, fixed ureteral narrowing, loss of renal function, and the need for reconstructive surgery (2–4).

Congenital Müllerian anomalies add a separate anatomic localization challenge. Reports on the use of IUDs in uterus didelphys show that the device position must be interpreted relative to two distinct uterine cavities (5–7). This becomes especially important when duplicated anatomy is recognized only after an IUD has already been placed or when later removal cannot be verified.

We report a case in which a uterus didelphys was unrecognized when an IUD was inserted and was first identified during evaluation of an unintended pregnancy 2−3 years later. This clinical episode included induced abortion and an IUD removal procedure, but the original records are no longer available to verify device location or intact retrieval. More than two decades later, an intact ectopic IUD was identified adjacent to a fixed distal ureteral stricture requiring reconstruction. The central clinical lesson is the prolonged verification gap created by presumed removal when duplicated uterine anatomy had only recently been recognized.

## 2 Case description

### 2.1 Patient information and clinical findings

A 48-year-old woman presented with a 1-year history of right flank pain and urinary frequency. She had received repeated treatment elsewhere for presumed urinary tract infection and possible genitourinary tuberculosis, but had not sustained improvement. More than 20 years ago, an IUD had been inserted at a local hospital; however, the original device model, manufacturer, and insertion details were unavailable. At insertion, uterus didelphys was not diagnosed, and the patient was unaware of the congenital uterine anomaly.

Two to three years after IUD insertion, she developed an unintended pregnancy and attended another hospital for induced abortion. Uterus didelphys was first identified during the evaluation of the pregnancy. Induced abortion and an IUD removal procedure were performed during the same episode. The original insertion, pregnancy, abortion, and removal records could not be retrieved. Therefore, the available evidence cannot establish which uterine cavity contained the pregnancy, where the IUD was located at that time, or whether intact retrieval was documented. The patient thereafter believed that the IUD had been removed; no surviving record documents imaging confirmation of device absence.

Apart from the gynecological history described above, the admission record described her as previously healthy and documented no known drug or food allergies, history of trauma, or history of transfusion. She reported no family history of hereditary disease or uterus didelphys and no history of psychosocial disorders. Genetic testing results were not available. Before admission, she had self-administered long-term oral levofloxacin obtained elsewhere for a presumed urinary tract infection (UTI). The dose and duration of levofloxacin use and details of other previous empiric regimens were unavailable.

At presentation, the temperature was 36.0 °C, pulse 69 beats/min, respiratory rate 20 breaths/min, and blood pressure 101/72 mmHg. She was alert and breathing comfortably. There was no renal-angle tenderness or percussion tenderness on either side, no tenderness along the ureteral courses, and no suprapubic distension or tenderness. Gynecological examination revealed no uterine or adnexal tenderness and no palpable adnexal mass.

The chronology is summarized in Table 1 and illustrated in Supplementary Figure S1. A CARE reporting checklist is provided in Supplementary Material S2.

**Table 1:** Clinical timeline.

Time	Clinical event
More than 20 years before index admission	IUD inserted at a local hospital. The uterus didelphys had not been diagnosed, and the original device model, manufacturer, and insertion records were unavailable.
2–3 years after insertion	An unintended pregnancy prompted evaluation. Uterus didelphys was first identified. Induced abortion and IUD removal were performed; however, surviving records did not establish device location or document intact retrieval.
Approximately 1 year before index admission	Right flank pain and urinary frequency developed. Repeated treatment for presumed urinary tract infection or possible genitourinary tuberculosis did not result in sustained improvement.
Index admission	The serum creatinine level was 53 μmol/L. Urinalysis showed pyuria with a negative urine culture, and tuberculosis-related testing did not support active genitourinary tuberculosis. Imaging revealed severe right hydroureteronephrosis, distal ureteral narrowing, and an ectopic metallic IUD outside both uterine cavities. The cystoscopy findings were unremarkable.
Surgery	An intact ectopic metallic ring-shaped IUD with an attached 0.5 × 0.6 cm calculus was removed. A persistent approximately 1-cm distal ureteral stricture required segmental resection and ureteral anastomosis over a double-J stent. Histopathology revealed chronic inflammatory ureteral changes without tuberculous changes.
2- and 6-month follow-up	The double-J ureteral stent was removed 2 months after surgery. At 6 months (4 months after stent removal), CT revealed no residual pelvic metallic device and marked improvement of right hydroureteronephrosis; serum creatinine was 50 μmol/L, and no procedure-related complication was documented.

### 2.2 Diagnostic assessment

At the index admission, serum creatinine was 53 μmol/L, within the local reference range. Urinalysis showed pyuria, whereas urine culture was negative. TB-DNA was below the assay detection limit, acid-fast staining was negative, and the interferon-gamma release assay was negative. Although the tuberculosis antibody result was weakly positive, the infectious disease consultation considered the available evidence insufficient to diagnose active genitourinary tuberculosis. Intravenous urography showed impaired right renal excretion and a metallic pelvic device. Computed tomography (CT) demonstrated severe right hydroureteronephrosis and focal narrowing of the distal right ureter adjacent to the metallic device. Three-dimensional transvaginal ultrasonography confirmed two uterine cavities and demonstrated an IUD-like echogenic structure outside both. Cystoscopy was unremarkable. Retrograde pyelography further localized the metallic ring adjacent to the distal right ureter. The radiographic and CT findings are shown in Figure 1. Together, the laboratory, imaging, and endoscopic findings favored a mechanical distal ureteral obstruction associated with an ectopic IUD rather than the previously presumed infectious causes.

**Figure 1:**
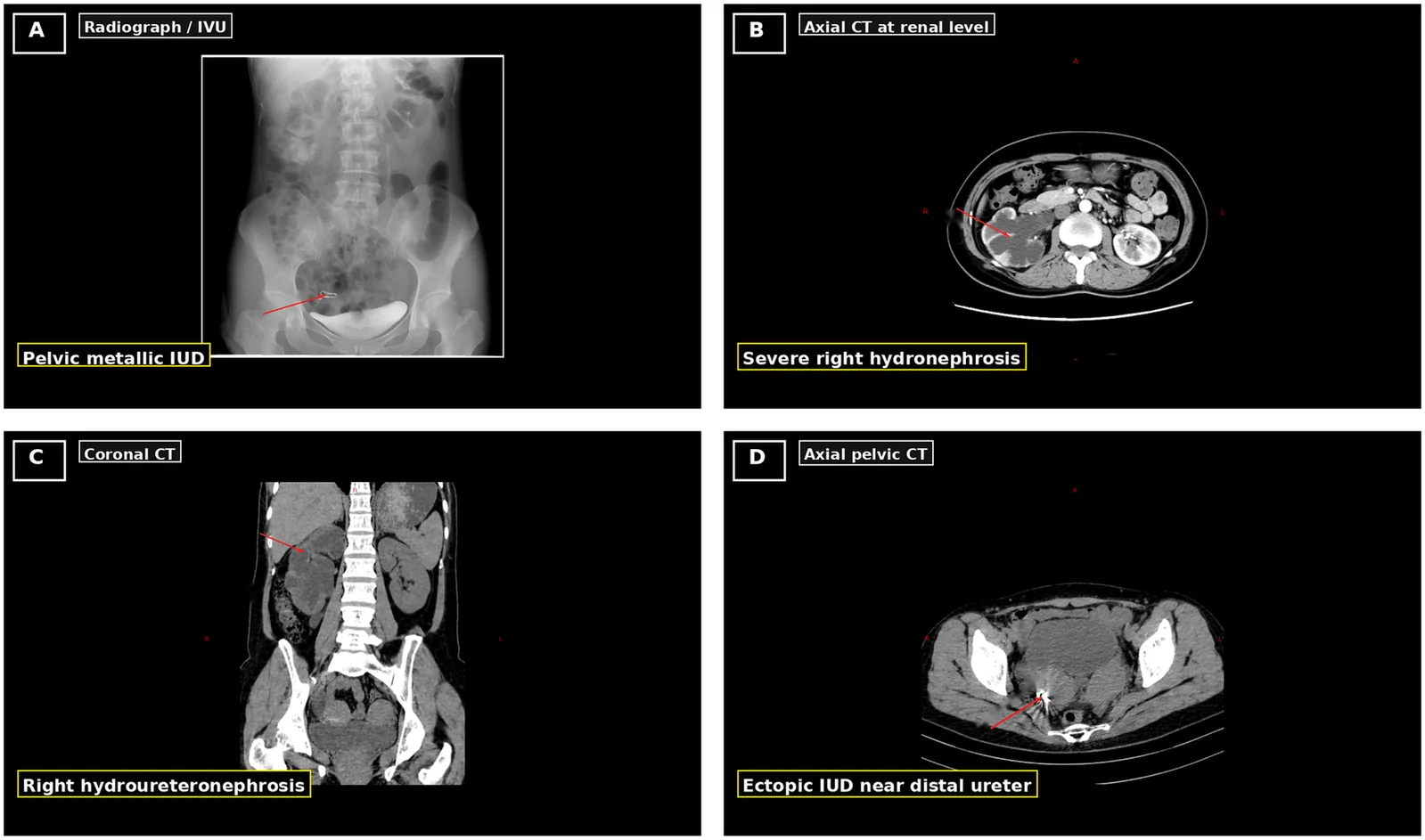
Preoperative imaging of right urinary tract obstruction and ectopic IUD. **(A)** Radiograph/intravenous urography demonstrates a radiopaque pelvic IUD (arrow). **(B)** Axial CT at the renal level shows severe right hydronephrosis (arrow). **(C)** Coronal CT shows marked right hydroureteronephrosis (arrow). **(D)** Axial pelvic CT demonstrates the ectopic metallic IUD immediately adjacent to the distal right ureter (arrow).

### 2.3 Therapeutic intervention

Open exploration under general anesthesia through a right lower abdominal extraperitoneal incision identified an approximately 1-cm fixed distal right ureteral stricture with marked proximal dilation. An ectopic metallic ring-shaped IUD was located outside the ureter and compressed it externally, without entering the ureteral lumen. The device, with an attached 0.5 × 0.6 cm calculus, was removed from the periureteral region. An intraoperative gynecological consultation confirmed that the retrieved device was intact and entirely extrauterine, without adhesions to the uterus. Its model, manufacturer, and material composition could not be reliably established. After device removal, the narrowing remained fixed, and ureteral patency was not restored. The stenotic segment was therefore resected. The proximal and distal ureteral segments were assessed with 6-Fr catheters and were found to be patent; ureteral anastomosis was then performed with interrupted 3-0 absorbable sutures over a 7-Fr double-J stent. Histopathologic examination of the resected segment showed chronic inflammatory changes without tuberculous changes. Postoperative care included levofloxacin and supportive treatment, and recovery was uneventful. Operative and retrieved-device photographs were not obtained at the time of surgery.

### 2.4 Follow-up and outcomes

The double-J ureteral stent was removed 2 months after the operation. At 6 months after surgery (4 months after stent removal), follow-up CT showed no residual pelvic metallic device and marked improvement in right hydroureteronephrosis compared to the preoperative study (Figure 2). Serum creatinine level was 50 μmol/L, which was within the normal range. No procedure-related complications were observed during the follow-up period.

**Figure 2:**
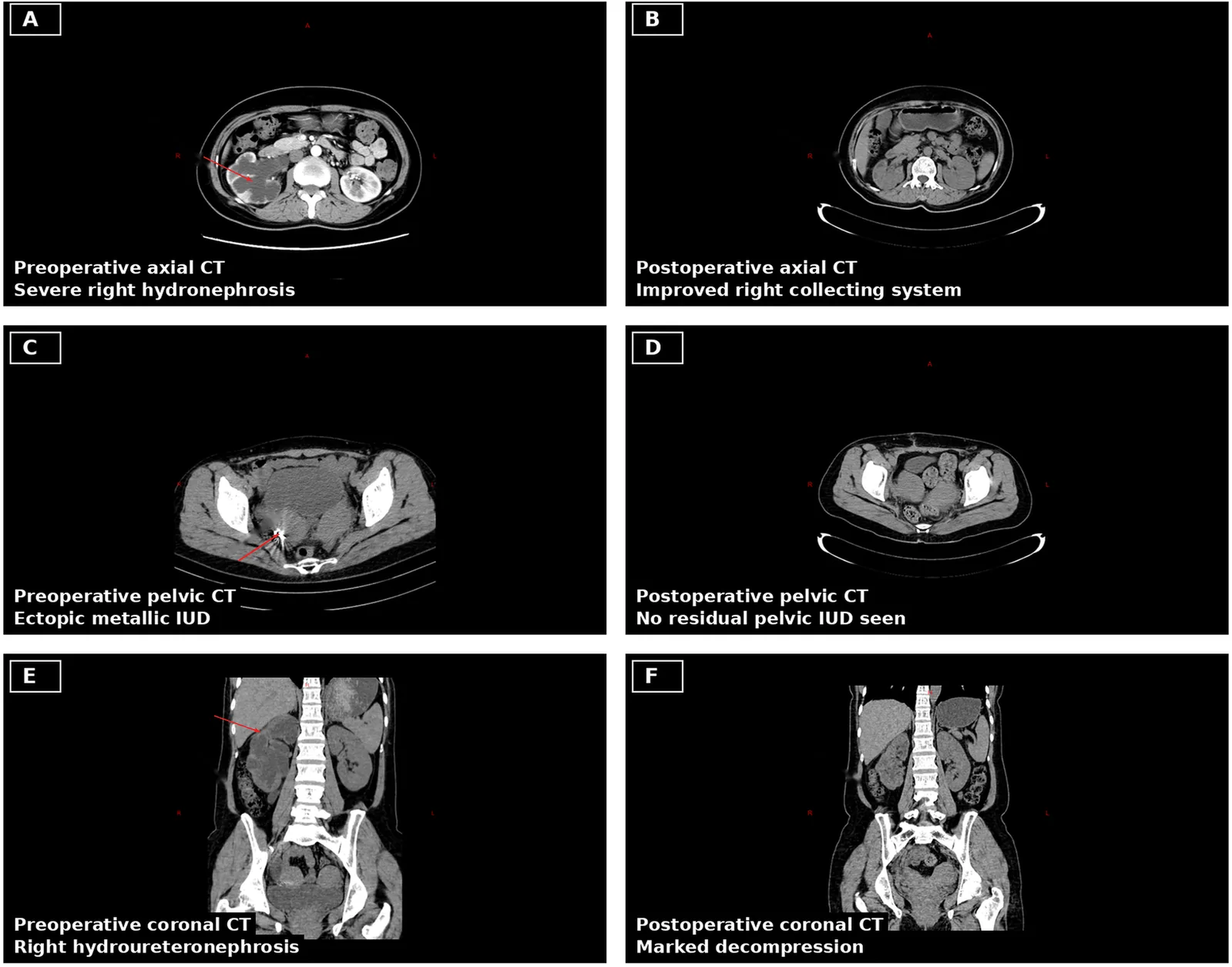
Preoperative and 6-month postoperative CT comparison. **(A, C, and E)** Representative preoperative axial renal, axial pelvic, and coronal CT images show severe right hydronephrosis/hydroureteronephrosis and ectopic pelvic IUD. **(B, D, and F)** Representative images obtained 6 months postoperatively and 4 months after double-J stent removal show marked decompression of the right collecting system and no residual pelvic IUD. The comparison demonstrates sustained anatomic improvement after IUD removal and ureteral reconstruction.

## 3 Discussion

### 3.1 Clinical interpretation in relation to prior reports

Previous reports have illustrated a spectrum of ureteral injuries caused by migrated IUDs. Yang et al. described worsening hydronephrosis after device removal and temporary double-J stenting, ultimately requiring ureterocystostomy (2). Karkin et al. reported severe periureteral fibrosis requiring ureteroneocystostomy and stenting (4), whereas Li et al. described delayed recognition with near-complete renal functional loss requiring nephrectomy (3). The 2024 review also showed that bladder involvement is far more common than ureteral involvement (1); in the present case, cystoscopy was unremarkable, arguing against concomitant intravesical involvement. Our patient underwent kidney-preserving ureteral reconstruction because the fixed distal stricture persisted after IUD removal and required segmental resection and anastomosis. Serum creatinine remained within the normal range, although this does not establish preserved function of the affected kidney because split renal function data were unavailable. Therefore, IUD-related ureteral obstruction is an established complication rather than a novel finding. The diagnostic problem in this case was that the device had been believed to be absent for more than two decades after a removal procedure, while the uterus didelphys was only recognized during that same historical episode. This verification gap persisted until current imaging reopened the possibility of an ectopic device during evaluation of the urinary obstruction.

In our patient, the IUD compressed the ureter externally without entering its lumen. The distal narrowing remained fixed after IUD removal, supporting an established ureteral stricture rather than transient external compression alone. Histopathology demonstrated chronic inflammatory ureteral changes without tuberculous changes, providing tissue-level evidence against the previously suspected tuberculous explanation. Together with the periureteral device location, unremarkable cystoscopy, persistent narrowing after extraction, successful stent removal at 2 months, and sustained decompression on the 6-month CT, the findings support a localized structural ureteral process associated with the ectopic IUD. However, these findings do not establish the timing or mechanism of extrauterine displacement or prove that the IUD alone caused the stricture. Therefore, when imaging or intraoperative assessment suggests a fixed lesion, the surgical plan should allow for reconstruction rather than assuming that extraction alone will restore ureteral patency.

Uterus didelphys should be interpreted here as an anatomic context rather than a proven cause of contraceptive failure, uterine perforation, or extrauterine displacement. Published experience with IUD use in uterus didelphys demonstrates why device position must be interpreted relative to two separate uterine cavities (5–7). Once duplicated anatomy is recognized, subsequent localization and removal verification should account for both cavities. In this case, however, the missing historical records preclude reconstruction of the device location during the pregnancy and removal episode and do not establish when or how the device became extrauterine. Conclusions about the mechanism must therefore remain limited.

The diagnostic lesson is directly supported by the available evidence: a history of “IUD removal” is not equivalent to a documented complete retrieval. When retrieval cannot be verified, ultrasonography or pelvic radiography can determine whether a radiopaque device remains, and cross-sectional imaging can define its relationship with adjacent organs (1). In otherwise unexplained unilateral hydroureteronephrosis, a remote contraceptive device history should remain part of the history review, especially when old records are unavailable or symptoms have prompted repeated empiric treatment for presumed infection.

### 3.2 Strengths and limitations

This report combines multimodality localization, unremarkable cystoscopy, operative confirmation of an approximately 1-cm fixed distal ureteral stricture that persisted after removal of the externally compressing IUD, and histopathology showing chronic inflammatory changes without tuberculous changes. Follow-up included double-J stent removal at 2 months and CT at 6 months after surgery, corresponding to 4 months after stent removal, which showed decompression of the right collecting system. The timeline distinguishes the remote patient-reported history from the current investigation and the treatment.

The principal limitation was the absence of historical insertion, pregnancy, abortion, and removal records. The exact time and mechanism of extrauterine displacement could not be determined. Operative and retrieved-device photographs were unavailable. The device model and composition could not be verified. The dose and duration of preadmission levofloxacin use and details of other previous empiric regimens were unavailable. Histopathology cannot determine when inflammation began or prove that the IUD alone caused the stricture. Normal serum creatinine does not exclude impaired ipsilateral function, and split renal function data were unavailable. The favorable CT result at 6 months after surgery provides only short-term anatomic follow-up; longer observation is needed to assess recurrent narrowing.

### 3.3 Take-away lessons

Three practical lessons have emerged from this study. First, when a uterus didelphys or another duplicated uterine anatomy is recognized after IUD placement, subsequent localization should evaluate both cavities. Second, complete removal should be documented, including the retrieval of an intact device; if retrieval cannot be verified, targeted imaging can prevent presumed removal from becoming a long-term diagnostic blind spot. Third, when an ectopic IUD lies adjacent to the ureter, clinicians should assess for fixed stricture because device removal alone may not relieve established obstruction.

## 4 Patient perspective

The patient reported that she had believed for more than two decades that the IUD had been removed and was unaware that a device might still be present. From her perspective, the earlier removal procedure resolved the device history until the current evaluation identified an ectopic IUD.

## Data Availability

The original contributions presented in the study are included in the article/Supplementary Material, further inquiries can be directed to the corresponding author.

## Supplementary material

The Supplementary Material for this article can be found online at: https://www.frontiersin.org/articles/10.3389/fmed.2026.1985408/full#supplementary-material

SUPPLEMENTARY FIGURE S1 Clinical timeline of reported IUD insertion and removal, current presentation, surgical treatment, and follow-up. Historical intervals are approximate and based on patient recall; the sequence is not drawn to scale. IUD, intrauterine device; CT, computed tomography.

SUPPLEMENTARY MATERIAL S2 Completed CARE checklist for this case report.
