# Supplementary material for: Case Report: Distal ureteral obstruction decades after presumed intrauterine device removal in a woman with uterus didelphys
Source: Front Med (Lausanne). 2026 Oct 7;13:1985408. doi: 10.3389/fmed.2026.1985408 (PMC13683640; doi:10.3389/fmed.2026.1985408)
Supplement: Supplementary file 1 [file Table1.docx]

Supplementary Material S2

# CARE reporting checklist

Manuscript 1985408. Adapted item-by-item mapping to the 2013 CARE checklist. Page numbers refer to the clean revised manuscript in this package. Missing or incompletely documented information is identified explicitly.

## Items 1 to 7

| **Item** | **Topic** | **Location** | **Reporting status** |
| --- | --- | --- | --- |
| 1 | Title | Title page Page 1 | Diagnosis and case-report designation included. |
| 2 | Keywords | Keywords Page 2 | Five terms, including case report. |
| 3a | Abstract contribution | Abstract Page 2 | Diagnostic problem after presumed removal. |
| 3b | Abstract presentation | Abstract Page 2 | Symptoms and principal imaging findings. |
| 3c | Abstract management | Abstract Page 2 | Diagnosis, reconstruction and follow-up. |
| 3d | Abstract lesson | Abstract Page 2 | Removal verification and associated stricture. |
| 4 | Introduction | Section 1 Page 2 | Context and prior ureteral cases. |
| 5a | Patient details | Section 2.1 Page 2–3 | Age and sex; no patient identifiers. |
| 5b | Presenting concerns | Section 2.1 Page 2–3 | Flank pain and urinary frequency. |
| 5c | Background history | Sections 2.1 and 3.2 Page 3, 5 | No family history of hereditary disease or uterus didelphys; no psychosocial disorders. Genetic testing results unavailable. Prior oral levofloxacin use described. |
| 5d | Previous interventions | Section 2.1 Page 3 | Historical procedures and long-term oral levofloxacin use described; historical records, dose and duration unavailable. |
| 6 | Clinical examination | Section 2.1 Page 3 | Vital signs and relevant examination findings. |
| 7 | Chronology | Table 1; Figure S1 Page 9–10 | Relative dates; recalled history distinguished. |

# CARE reporting checklist continued

## Items 8 to 10

| **Item** | **Topic** | **Location** | **Reporting status** |
| --- | --- | --- | --- |
| 8a | Diagnostic methods | Section 2.2 Page 3 | Laboratory tests, imaging and endoscopy. |
| 8b | Diagnostic difficulties | Sections 2.1–2.2 and 3.2 Page 3, 5 | Unavailable historical records and prior presumptive infection diagnoses. |
| 8c | Diagnosis and alternatives | Sections 2.2–2.3 Page 3–4 | Distal stricture and ectopic IUD; infection and tuberculosis considered. |
| 8d | Prognostic assessment | Sections 2.4 and 3.2 Page 4–5 | Creatinine and anatomic follow-up; split renal function data unavailable. Tumor staging not applicable. |
| 9a | Treatment modalities | Section 2.3 Page 3–4 | Open retrieval, resection, anastomosis and postoperative care. |
| 9b | Treatment details | Sections 2.3–2.4 Page 3–4 | Catheter/stent sizes, suture and stent duration. Levofloxacin dose/duration unavailable. |
| 9c | Treatment changes | Sections 2.1–2.3 Page 3–4 | Prior empiric treatment followed by surgical management of obstruction. |
| 10a | Clinical outcomes | Section 2.4 Page 4 | Uneventful recovery and postoperative imaging. No formal patient-reported outcome measure. |
| 10b | Follow-up tests | Section 2.4; Figure 2 Page 4, 8 | CT and creatinine at 6 months after surgery; original Figure 2 supplied and included. |
| 10c | Adherence and tolerance | Sections 2.3–2.4 Page 3–4 | Stent removal and follow-up documented; no formal medication-adherence assessment. |
| 10d | Adverse events | Section 2.4 Page 4 | No procedure-related complications documented during reported follow-up. |

# CARE reporting checklist continued

## Items 11 to 13

| **Item** | **Topic** | **Location** | **Reporting status** |
| --- | --- | --- | --- |
| 11a | Strengths and limits | Section 3.2 Pages 4–5 | Records, imaging, causal and follow-up limitations. |
| 11b | Literature context | Section 3.1; references 1–7 Page 4–6 | Previous urinary-tract IUD cases discussed. |
| 11c | Interpretation | Sections 3.1–3.2 Page 4–5 | Association distinguished from proven causation. |
| 11d | Clinical lessons | Section 3.3 Page 5 | One-paragraph practical lessons. |
| 12 | Patient perspective | Section 4 Page 5 | Belief in prior removal reported; postoperative treatment perspective not documented. |
| 13 | Publication consent | Ethics statement Page 5 | Written informed consent obtained from the patient for publication of this case report and accompanying images. |

Checklist source: CARE Case Report Guidelines, 2013. https://www.care-statement.org/checklist (accessed 20 September 2026).
